# Population Pharmacokinetic Modeling of Zaltoprofen in Healthy Adults: Exploring the Dosage Regimen

**DOI:** 10.3390/ph16020161

**Published:** 2023-01-22

**Authors:** Ji-Hun Jang, Seung-Hyun Jeong, Yong-Bok Lee

**Affiliations:** 1 College of Pharmacy, Chonnam National University, 77 Yongbong-ro, Buk-gu, Gwangju 61186, Republic of Korea; 2 College of Pharmacy, Sunchon National University, 255 Jungang-ro, Suncheon-si 57922, Republic of Korea

**Keywords:** zaltoprofen, population pharmacokinetic modeling, dosage regimen, creatinine clearance, albumin, CYP2C9

## Abstract

Zaltoprofen is a drug used for various pain and inflammatory diseases. Scientific and quantitative dosage regimen studies regarding its clinical application are scarce. This study aimed to discover effective covariates related to interindividual pharmacokinetic variability through population pharmacokinetic modeling for zaltoprofen and to explore dosage regimens. The bioequivalence results of healthy Korean males, biochemical analysis, and CYP2C9 genotyping information were utilized in modeling. The established model has been sufficiently verified through a bootstrap, goodness-of-fit, visual predictive check, and normalized prediction distribution error. External data sets derived from the literature were used for further model validation. The final model could be used to verify the dosage regimen through multiple exposure simulations according to the numerical change of the selected covariates. Zaltoprofen pharmacokinetics could be explained by a two-compartment with a first-order absorption model. Creatinine clearance (CrCL) and albumin were identified as effective covariates related to interindividual zaltoprofen pharmacokinetic variability, and they had positive and negative correlations with clearance (CL/F), respectively. The differences in pharmacokinetics between individuals according to CYP2C9 genetic polymorphisms (*1/*1 and *1/*3) were not significant or valid covariates. The model simulation confirmed that zaltoprofen pharmacokinetics could significantly differ as the CrCL and albumin levels changed within the normal range. Steady-state plasma exposure to zaltoprofen was significantly reduced in the group with CrCL and albumin levels of 130 mL/min and 3.5 g/dL, respectively, suggesting that dose adjustment may be necessary. This study is useful to guide precision medicine of zaltoprofen and provides scientific quantitative judgment data for its clinical applications.

## 1. Introduction

Zaltoprofen is a nonsteroidal anti-inflammatory drug widely used for various inflammatory and painful diseases such as rheumatoid arthritis, osteoarthritis, toothache, and complex regional pain syndrome [1,2,3]. Zaltoprofen reduces the production of prostaglandins, an inflammatory substance, through the inhibition of cyclooxygenase (COX) [4,5,6], and exerts analgesic action by strongly inhibiting bradykinin-induced nociceptive responses [4,7,8]. The general dose of zaltoprofen is 80–160 mg at a time, and it can be orally administered up to 240 mg per day depending on symptom severity [1,9]. The main clinical side effects of zaltoprofen include gastrointestinal bleeding and ulceration, irritability, and psychosomatic disorders, such as headache and dizziness [9,10]. Gastrointestinal bleeding and ulceration caused by excessive COX suppression are the most significant side effects in the clinical application of zaltoprofen [10,11] and require drug monitoring.

Although zaltoprofen was officially approved in 1993 [12], pharmacokinetic and pharmacodynamic information is lacking. The quantitative prediction of pharmacokinetics for zaltoprofen and studies on the variability of pharmacokinetics between individuals within a population are very scarce. Despite the active clinical application of zaltoprofen [13], the clinical dosage regimen depends on empirical knowledge and observed side effects. Scientific data on the management of side effects and risk and efficacy prediction of zaltoprofen is lacking.

Pharmacokinetic variability within a group and drug modeling studies are useful scientific approaches to reducing side effects and maximizing drug efficacy by finding effective dosages based on quantitative predictions [14,15,16]. The purpose of this study was to perform population pharmacokinetic (PPK) modeling of zaltoprofen and discover effective covariates affecting interindividual pharmacokinetic variability. Via model simulation, we attempted to predict the degree of pharmacokinetic change based on the changes in the covariates and set the dosage regimen. This study is important as it identified the PPK properties of zaltoprofen and provided useful quantitative information for clinical application.

## 2. Results

### 2.1. Population Pharmacokinetic Modeling

The basic structure of the zaltoprofen PPK model can be explained by two compartments with first-order absorption. In structures of three-compartment or higher, the decrease in the negative log-likelihood (-2LL) was not significant compared to the increase in the total number of parameters. Several structural multiple absorption compartment models, such as nonsequential two-absorption compartments (having two or more absorption points with bioavailability considerations) and sequential two-absorption compartments (two or more absorption rate constant parameters between successive absorption compartments), were tried with respect to the plasma concentration profile in the absorption phase. No significant model improvement was noted. The reflection of lag-time during absorption increased the value of -2LL along with the increased number of model parameters. Therefore, the zaltoprofen absorption process was described without adding a separate mechanistic structure or reflecting the lag time.

The proportional error model was most suitable as the residual error model. When applied, the reduction of -2LL was significantly greater while the total number of parameters compared to other error models was maintained. The IIVs in pharmacokinetic parameters of zaltoprofen were evaluated using an exponential error model, as shown in the following equation: P_i_ = P_tv_ · exp(*ŋ*_i_), where *ŋ*_i_ is the random variable for the i^th^ individual, which was normally distributed with a mean 0 and variance *ω*^2^; P_i_ is the parameter value of the ith individual; and P_tv_ is the typical value of the population parameter. After checking the necessity of considering IIV in each parameter step-wise for model improvement, only IIV was considered for V_2_/F, CL/F, and K_a_. Here, V_2_ and CL are the mean volume and clearance in the peripheral and central compartments, respectively. K_a_ and F were the oral absorption rate constant and bioavailability (of oral administration), respectively. Table S1 summarizes the steps for establishing a basic model structure for zaltoprofen.

Genetic polymorphisms of CYP2C9 and several biochemical factors were considered as candidate covariates to explain the interindividual pharmacokinetic variability of zaltoprofen. The covariates were screened based on non-compartment analysis (NCA) results before model reflection. Figure S1 shows the results of comparing T_1/2_, T_max_, C_max_, AUC, V/F, and CL/F according to the CYP2C9 genotype. It was confirmed that 22 individuals had the CYP2C9*1/*1 genotype and four had the CYP2C9*1/*3 genotype. No significant difference was found between CYP2C9*1/*1 and *1/*3 in any of the parameters except T_max_. As shown in Figure S2, no significant differences were found between groups in the plasma concentration profiles observed between CYP2C9*1/*1 and *1/*3. Correlation analysis between biochemical factors and pharmacokinetic parameters revealed that CrCL-CL/F showed a high positive correlation with *R^2^* 0.245, while albumin-CL/F showed a high negative correlation with *R^2^* 0.158. Correlation graphs between biochemical factors and pharmacokinetic parameters, which had a correlation of more than approximately 30%, are presented in Figure S3. Results confirmed to have a relatively low correlation of 30% or less during the screening process are presented in Figure S4. These were primarily excluded from the model covariate application process.

Finally, to explain the interindividual pharmacokinetic variability of zaltoprofen, CrCL and albumin levels were considered effective covariates with respect to CL/F. This model reflection significantly (based on *p* < 0.05 and *p* < 0.01 for forward selection and backward elimination, respectively) improved the model. The reflection of covariates in CYP2C9 genotypes did not significantly improve the model. The covariate reflection results of the CYP2C9 genotypes for CL/F met the forward selection criteria but were not significant in the backward elimination process. Moreover, no significant model visual improvement was found in the goodness-of-fit (GOF) plot results. Table S2 summarizes the model reflection steps and results of the CYP2C9 genetic polymorphism and biochemical factors considered as potential covariates in the established basic PPK model parameters for zaltoprofen. The formulas for the finally established PPK model parameters of zaltoprofen were as follows:

(1) $$\frac{V}{F}=\mathrm{tv}\;\frac{V}{F}$$

(2) CLF=tv CLF · (CrCL/m CrCL)^dCL/FdCrCL · (Albumin/m Albumin)^dCL/FdAlbumin · exp(ηCL/F) 

(3) $$\frac{V_{2}}{F}=\mathrm{tv}\;\frac{V_{2}}{F}\;\cdot \;\exp(\eta V_{2}/F)\;$$

(4) $$\frac{{\mathrm{CL}}_{2}}{F}=\mathrm{tv}\;\frac{{\mathrm{CL}}_{2}}{F}$$

K_a_ = tv K_a_ · exp(*ŋ*_Ka_) (5)

where tv and m are the typical and median values, respectively, V and CL_2_ indicate volume and clearance in the central and peripheral compartments, respectively, dCL/FdCrCL is the degree of correlation between CL/F and CrCL, and dCL/FdAlbumin indicates the degree of correlation between CL/F and albumin. The parameters of the finally established PPK model of zaltoprofen and their related values are presented in Table S3. The relative standard errors (RSEs) of typical values of the pharmacokinetic parameters K_a_, V/F, CL/F, V_2_/F, and CL_2_/F were within 30%, demonstrating sufficient reasonable agreement (Table S3).

### 2.2. Model Qualification

The GOF plot results of the zaltoprofen PPK model established in this study are presented in Figure S5. Zaltoprofen concentration values predicted by the PPK model at the population or individual levels showed relatively good agreement with the experimentally obtained observations. The conditional weighted residuals (CWRES) were well distributed symmetrically with respect to zero. That is, CWRES were well distributed at random without any remarkably specific bias. The CWRES values at all points of predicted concentrations or time in the population did not deviate from ± 4. Quantile–quantile (QQ) plots of CWRES and individual weighted residuals (IWRES) were close to a straight line, where the X- and Y-axes were symmetrical (within ± 6 ranges). Consequently, the GOF plot results (Figure S5) suggest that the final established PPK model had no graphically significant problems. Bootstrapping results for the established zaltoprofen PPK model are presented in Table S4. All of the parameter values estimated in zaltoprofen’s final model were within the 95% confidence interval of the bootstrap analysis results (1000 replicates). The estimates of the model parameters were close to the median estimated by bootstrapping, with differences within 20%.

Bootstrapping analysis confirmed the robustness and reproducibility of the final established PPK model of zaltoprofen. The normalized prediction distribution error (NPDE) analysis results are presented in Figure S6. The assumption of a normal distribution for the differences between predictions and observations of zaltoprofen pharmacokinetics was acceptable. The QQ plots and histogram also confirmed the normality of the NPDE. Moreover, the NPDE results over time and predicted values were relatively symmetric with respect to zero (within ± 4 ranges). The visual predictive check (VPC) result of the zaltoprofen PPK model is presented in Figure S7. Most of the observation values (>90% of all data) of zaltoprofen pharmacokinetics were well distributed within the 95% confidence intervals of the prediction values. The VPC results suggest that zaltoprofen’s PPK model described the overall experimental data relatively well. The final established PPK model of zaltoprofen was at an acceptable level in the overall evaluation results with no major problems.

### 2.3. External Model Validation

**Figure 1:**
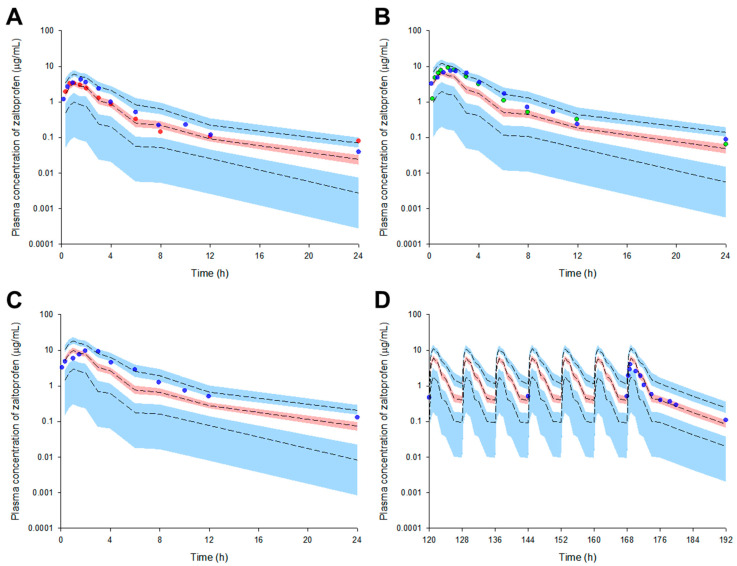
Simulation graphs of the pharmacokinetics of the final model for zaltoprofen according to single dose change ((**A**) 80 mg, (**B**) 160 mg, and (**C**) 240 mg) or multiple doses ((**D**) 80 mg). Observed concentrations after oral administration of 80–240 mg are depicted by the colored dots. The presented multiple exposure graph (**D**) is the simulation result for the observation area (120–192 h). The 95th, 50th, and 5th percentiles of the predicted concentrations are represented by black dashed lines. The 95% confidence intervals (CI) for the predicted 5th and 95th percentiles are represented by the blue-shaded regions. The 95% CI for the predicted 50th percentiles is represented by the red-shaded regions. The X-axis represents the time after oral zaltoprofen administration and the Y-axis is the observed and predicted zaltoprofen concentrations in the plasma. The red (**A**), blue (**A**–**D**), and green (**B**) dots represent mean data from Kang et al., (2006), Li et al., (2011), and Lee et al., (2006), respectively.

We externally validated the model to confirm that it can be reasonably applied to external data sets obtained after various dosage regimens. The structure and parameters of the zaltoprofen PPK model established in the previous process were maintained, and the dose and dosing interval were changed to fit the external validation data set. The VPC results of the model simulation are presented in Figure 1. Most of the reported observations (>90% of all data) related to the zaltoprofen pharmacokinetics were well distributed within the 95% confidence interval of the prediction values. This suggests that the PPK model of zaltoprofen established in this study could reasonably explain other data at the same dose (80 mg) and data in the groups with doses increased to 160 and 240 mg. Moreover, considering that steady-state observations according to the change in zaltoprofen dosing interval (8 h) are well predicted by the model, the model was reasonably accessible to the pharmacokinetic predictions according to multiple exposure simulations.

### 2.4. Exploring the Simulation-Based Dosage Regimen

Model simulations were performed according to the numerical changes in effective covariates selected from the final established zaltoprofen PPK model. That is, model simulations were performed assuming the maximum or minimum values of CrCL (80–130 mL/min) [17] or albumin (3.5–5.5 g/dL) [18] in the normal range for CrCL and albumin determined as effective covariates of CL/F. The zaltoprofen PPK model in this study was established based on the pharmacokinetic data of normal healthy adults [19]. In the simulation of single oral administration of zaltoprofen, the plasma concentration of the drug was higher and longer lasting in the group with CrCL levels of 80 mL/min compared to 130 mL/min and albumin levels of 5.5 g/dL compared to 3.5 g/dL. Moreover, plasma concentrations of zaltoprofen in the groups with CrCL and albumin levels of 80 mL/min and 5.5 g/dL, respectively, were higher and persisted for longer than those in the CrCL 80 mL/min or albumin 5.5 g/dL groups. 

**Figure 2:**
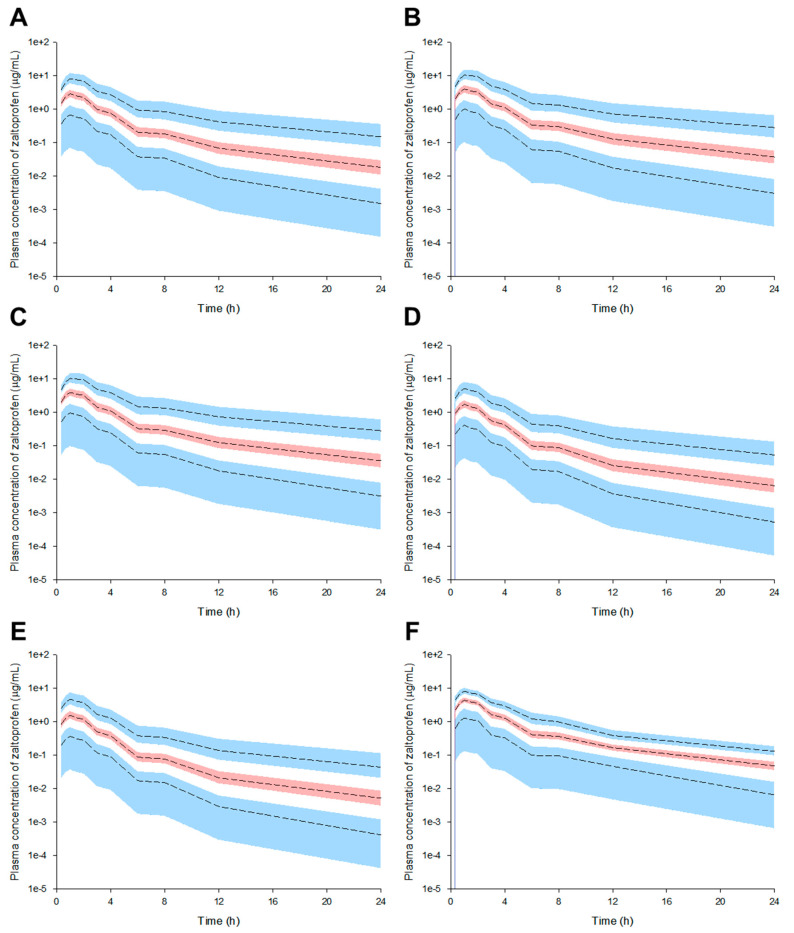
Pharmacokinetic prediction profiles after single oral administration of zaltoprofen (80 mg) according to the change in CrCL and albumin levels ((**A**) CrCL 130 mL/min, (**B**) CrCL 80 mL/min, (**C**) Albumin 5.5 g/dL, (**D**) Albumin 3.5 g/dL, (**E**) CrCL 130 mL/min and albumin 3.5 g/dL, and (**F**) CrCL 80 mL/min and albumin 5.5 g/dL). CrCL 130 and 80 mL/min and albumin 5.5 and 3.5 g/dL were randomly assigned to the upper and lower limits of the general range of healthy adults, respectively. The 95th, 50th, and 5th percentiles of the predicted concentrations are represented by black dashed lines. The 95% confidence intervals (CI) for the predicted 5th and 95th percentiles are represented by the blue-shaded regions. The 95% CI for the predicted 50th percentile is represented by the red-shaded regions. The X-axis indicates the time after oral zaltoprofen administration and the Y-axis is the predicted concentrations of zaltoprofen in the plasma.

The plasma concentrations of zaltoprofen in the group with CrCL and albumin levels of 130 mL/min and 3.5 g/dL, respectively, were lower and more quickly eliminated than those in the group with CrCL and albumin levels of 80 mL/min and 5.5 g/dL, respectively. Here, the numerical combinations of CrCL and albumin were randomly selected to show the differences between groups according to the model simulation. Figure 2 shows the simulation results of a single oral administration of zaltoprofen according to changes in CrCL and/or albumin levels.

**Figure 3:**
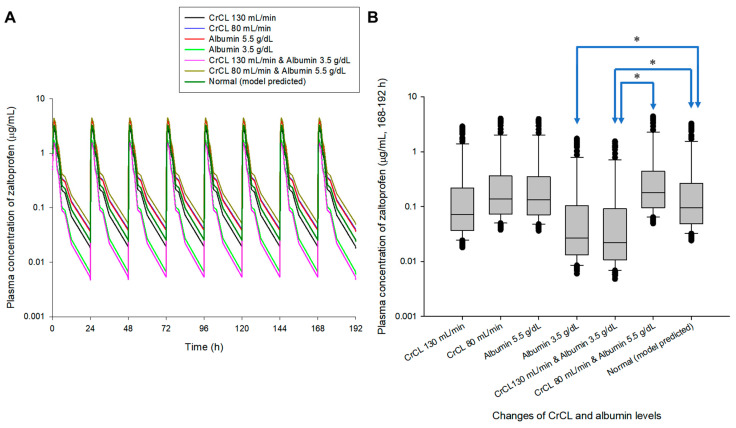

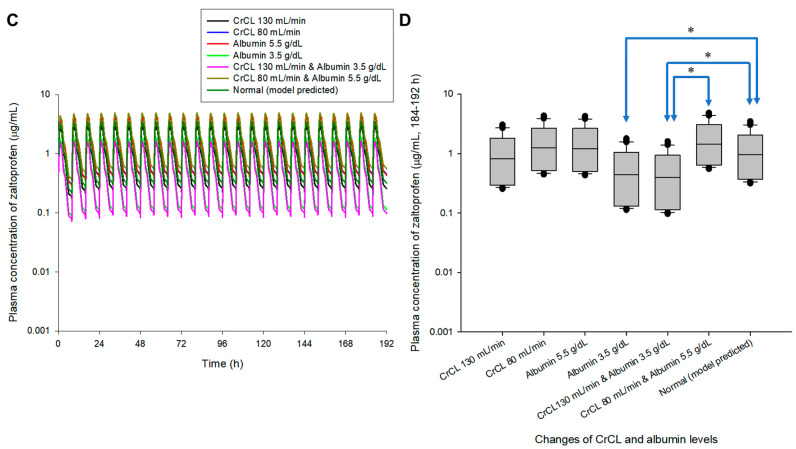
Simulation of pharmacokinetic prediction profiles after multiple (24 h (**A**,**B**) and 8 h intervals (**C**,**D**)) oral administrations of zaltoprofen (80 mg) according to the change in CrCL and albumin levels. The lines in the graph (**A**,**C**) indicate the mean values corresponding to 50% of each group. Normal in the graph is the 50% average predicted by the model established in this study (based on the group with median values of CrCL and albumin of 104.38 mL/min and 4.90 g/dL, respectively). A comparison (**B**,**D**) of predicted intergroup plasma concentrations at steady-state (168–192 h (**B**) and 184–192 h (**C**)) following changes in CrCL and albumin levels. * indicates a statistically significant difference between groups with *p* < 0.05.

Multiple exposure simulations were performed for the usual dosing regimen of zaltoprofen, 80 mg once daily or three times a day [1,9]. As a result of multiple oral administration simulations, the pharmacokinetic profile and steady-state mean plasma concentrations of zaltoprofen differed according to the changes in CrCL and albumin levels. Steady-state mean plasma concentrations in the CrCL 130 and 80 mL/min groups were 0.87 and 1.30 times that of the normal group, respectively. Here, the normal group had CrCL and albumin levels of 104.38 mL/min and 4.90 g/dL, respectively, meaning the group with median values of CrCL and albumin in the healthy adult group was used in this study. Steady-state mean plasma concentrations in the group with albumin levels of 5.5 and 3.5 g/dL were 1.28 and 0.48 times that of the normal group, respectively. The mean plasma concentrations in the groups with CrCL and albumin levels of 130 mL/min and 3.5 g/dL and 80 mL/min and 5.5 g/dL were 0.43 and 1.49 times those of the normal group, respectively. Quantitative comparative results of simulations of oral exposure to zaltoprofen at 8 h dosing intervals were also equivalent. As a result, it was suggested that the mean plasma exposure to zaltoprofen at steady-state could be significantly increased as the levels of CrCL decreased and the albumin levels increased. Figure 3 shows the comparison of pharmacokinetic profiles and steady-state predicted plasma concentration values for multiple exposures of zaltoprofen at 80 mg at 24 h and 8 h intervals according to changes in CrCL and albumin levels. A significant difference was confirmed between the normal group and the groups with albumin levels of 3.5 g/dL or CrCL levels of 130 mL/min in steady-state predicted plasma concentration values. A significant difference was also confirmed between the group with CrCL levels of 130 mL/min and albumin levels of 3.5 g/dL and those with CrCL levels of 80 mL/min and albumin levels of 5.5 g/dL. Zaltoprofen dose adjustment simulations were performed for the group with the highest and lowest zaltoprofen steady-state mean plasma concentrations of CrCL (80 mL/min) and albumin (5.5 g/dL) and CrCL (130 mL/min) and albumin (3.5 g/dL) (Figure 3).

**Figure 4:**
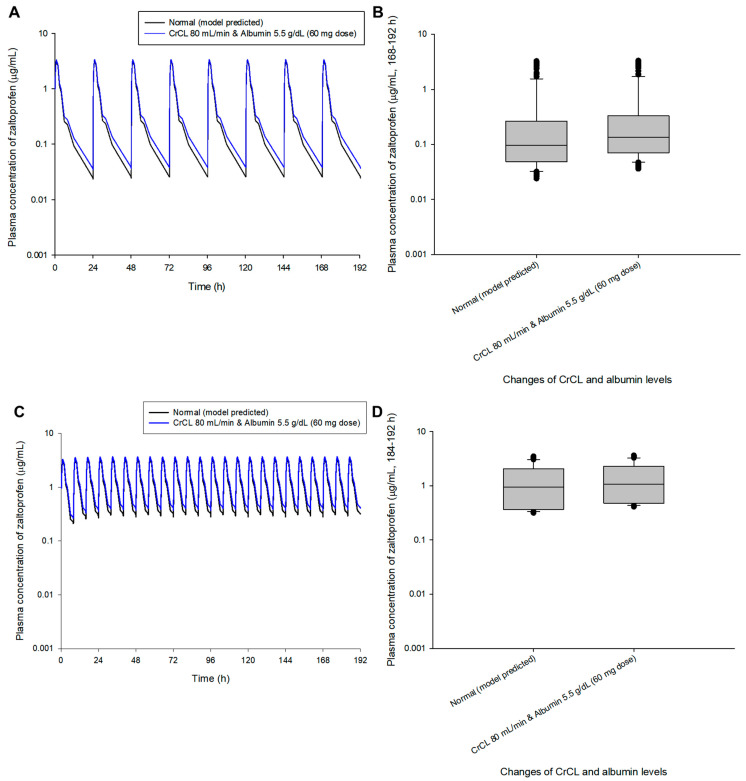
Simulation of the pharmacokinetic prediction profile after multiple (24 h (**A**,**B**) and 8 h intervals (**C**,**D**)) oral administrations of zaltoprofen predicted by arbitrarily adjusting the dose to 60 mg for the group with CrCL and albumin levels of 80 mL/min and 5.5 g/dL, respectively. The lines in the graph (**A**,**C**) indicate the mean values corresponding to 50% of each group. Normal in the graph indicates the 50% average predicted by the model established in this study (based on the group with median values of CrCL and albumin of 104.38 mL/min and 4.90 g/dL, respectively). Comparison (**B**,**D**) of predicted intergroup plasma concentrations at steady-state (168–192 h (**B**) and 184–192 h (**C**)) following a dose change of zaltoprofen to 60 mg for a group with CrCL and albumin levels of 80 mL/min and 5.5 g/dL, respectively.

**Figure 5:**
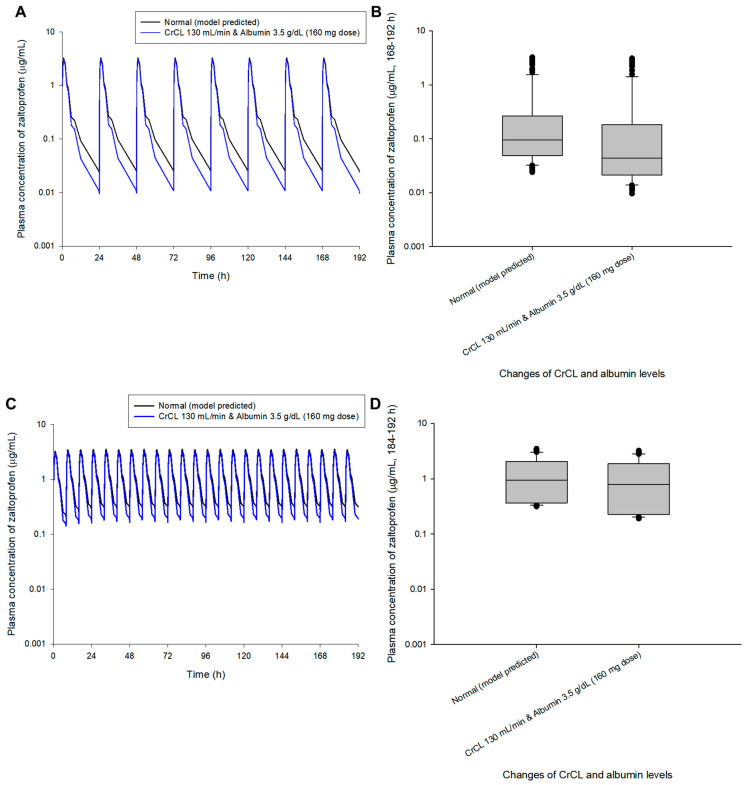
Simulation of the pharmacokinetic prediction profile after multiple (24 h (**A**,**B**) and 8 h intervals (**C**,**D**)) oral administrations of zaltoprofen predicted by arbitrarily adjusting the dose to 160 mg for the group with CrCL and albumin levels of 130 mL/min and 3.5 g/dL, respectively. The lines in the graph (**A**,**C**) indicate the mean values corresponding to 50% of each group. Normal in the graph indicates the 50% average predicted by the model established in this study (based on the group with median values of CrCL and albumin of 104.38 mL/min and 4.90 g/dL, respectively). Comparison (**B**,**D**) of predicted intergroup plasma concentrations at steady-state (168–192 h (**B**) and 184–192 h (**C**)) following a dose change of zaltoprofen to 160 mg for a group with CrCL and albumin levels of 130 mL/min and 3.5 g/dL, respectively.

A dosage adjustment was performed randomly through model simulation to approximate normal steady-state mean plasma concentrations (zaltoprofen at 80 mg in multiple doses at 24 h and 8 h intervals). Figure 4 shows the comparison of pharmacokinetic profiles and steady-state predicted values in the normal group after changing the zaltoprofen multiple-exposure dose from 80 to 60 mg in the group with CrCL levels of 80 mL/min and albumin levels of 5.5 g/dL. Reducing the dose to 60 mg in this group confirmed that the difference in the average concentration from the normal group at steady-state was reduced from 1.49 times (0.43 to 0.65 μg/mL) to 1.12 times (0.43 to 0.48 μg/mL). Figure 5 shows the comparison of pharmacokinetic profiles and steady-state predicted values in the normal group after changing the zaltoprofen multiple exposure dose from 80 to 160 mg in the group with CrCL levels of 130 mL/min and albumin levels of 3.5 g/dL. By increasing the zaltoprofen dose to 160 mg in this group, confirmed that the difference in the average concentration from the normal group at steady-state was reduced from 0.43 times (0.43 to 0.19 μg/mL) to 0.86 times (0.43 to 0.37 μg/mL). Simulated quantitative comparative results at the 8 h dosing interval of zaltoprofen were also identical to those at the 24 h dosing interval (Figure 4 and Figure 5).

## 3. Discussion

In this study, the genetic polymorphism information of CYP2C9 was prioritized as a reflection of covariates concerning the interindividual zaltoprofen pharmacokinetic variability. This was because CYP2C9 was confirmed to be involved in the zaltoprofen metabolism in a previous in vitro report [20], although information on genetic polymorphisms had not been reported. The clinical pharmacokinetics and CYP2C9 genotyping of the 26 subjects obtained in this study showed no significant differences in zaltoprofen pharmacokinetics according to genotype. By reflecting the genetic polymorphisms of CYP2C9 as covariates of CL/F (based on Table S2), model reconfirmation was performed with the following formula:

(6) CLF=tv CLF · [1+dCL/FdCYP2C9*1/*3 · (CYP2C9*1/*3=1)] · [(CrCL/m CrCL)^dCL/FdCrCL (optional according to CrCL covariate reflection)] · [(Albumin/m Albumin)^dCL/FdAlbumin (optional according to albumin covariate reflection)] · exp(ηCL/F) 

where dCL/FdCYP2C9*1/*3 indicates the degree of correlation of CL/F according to CYP2C9*1/*3.

Considering the CYP2C9 genetic polymorphism as a CL/F covariate, the estimated dCL/FdCYP2C9*1/*3 values were −0.09 to −0.07, and the median values according to bootstrapping also corresponded to −0.10 to −0.08. Therefore, the effect of the genetic polymorphism of CYP2C9*1/*3 on zaltoprofen pharmacokinetics would not be large (with CL/F differences within approximately 10%). Figure S8 shows VPC graphs for CYP2C9*1/*1 and *1/*3 obtained after reflecting the CYP2C9 genetic polymorphism as a covariate of CL/F. Between CYP2C9*1/*1 and *1/*3, the group mean predicted values corresponding to 50% were not significantly different visually. 

Data limitations may be related to the failure of CYP2C9′s genetic polymorphism to be selected as an effective covariate for zaltoprofen’s CL/F in this study. That is, pharmacokinetic and genotype information must be obtained for a larger population in the future, and the pharmacokinetic results for CYP2C9*3/*3 will be particularly important because zaltoprofen pharmacokinetic differences that were not evident in the heterogenic trait of CYP2C9*1/*3 may be very large in the homogenic CYP2C9*3/*3. This study was significant in that it scientifically suggested that zaltoprofen pharmacokinetic differences between CYP2C9*1/*1 and *1/*3 were small and that it is not essential to consider the genotype of CYP2C9 for clinical applications. This was predicted when considering the proportion of *3/*3 in the total population, which is approximately 1–2% [21], and the differences in pharmacokinetics in *1/*3 are proportional (although the pharmacokinetics of zaltoprofen for CYP2C9*3/*3 have not been confirmed experimentally).

Interestingly, CrCL and albumin were explored as effective covariates of CL/F, although the values of the biochemical parameters applied in this study were derived from a healthy adult population and were within the normal range. Table S5 presents the demographic information of the study subjects. Even though the CrCL and albumin levels had relatively narrow values of 107.53 ± 17.28 mL/min and 4.92 ± 0.18 g/dL, respectively, they were identified as CL/F covariates, suggesting that a large CL/F difference could occur in the patient group with abnormal values of CrCL and/or albumin. Patients with very low CrCL or very high albumin levels would have a significantly lower CL/F of zaltoprofen, which could cause the drug to accumulate in the body, possibly causing serious side effects. Therefore, the CrCL and albumin covariates discovered in this study will be useful as indirect data for setting the zaltoprofen dose in healthy adults and patients with specific conditions, such as kidney or liver disease. However, since this study is based on clinical data-based pharmacokinetic modeling and interpretation of results for a healthy adult population, direct application to actual patient groups will be limited. Therefore, it will be necessary to additionally secure clinical data for actual patient groups and verify models based on them in the future.

In the zaltoprofen PPK model established in this study, IIVs of K_a_ and V_2_/F were 40.63 and 48.55%, respectively (Table S3). This suggested that the variability in the absorption and distribution of zaltoprofen in the body is unaccounted for, with additional latent covariates. In particular, the function of transporters in drug absorption and distribution will be important, and polymorphisms of organic anion-transporting polypeptides (OATPs) may be involved [22]. In the future, in vitro testing of zaltoprofen substrates for several transporters and further analysis of pharmacokinetic correlations according to genetic polymorphisms will be required.

In the simulation of a single oral administration of zaltoprofen using the model established in this study, the plasma concentration rapidly decreased as CrCL levels increased or albumin levels decreased. Moreover, in the simulation of multiple oral administrations of zaltoprofen, the steady-state mean plasma concentration was lower as CrCL increased or albumin decreased. This is likely because CrCL and albumin levels had positive and negative correlations in zaltoprofen CL/F variability between individuals, respectively, and were reflected in dCL/FdCrCL and dCL/FdAlbumin in the model. The positive dCL/FdCrCL result was sufficiently convincing as a covariate correlation that renal excretion is one of the major factors in the clearance of zaltoprofen [20,23], and therefore CL/F increases as CrCL increases. For negative dCL/FdAlbumin, as the plasma protein binding rate of zaltoprofen was as high as 99.6% [20,24], an increase in albumin, a major plasma protein, is related to a decrease in the CL/F of zaltoprofen. That is, an increase in albumin may lead to a decrease in the concentration of unbound zaltoprofen, which may decrease CL/F and, consequently, an increase in the plasma concentration of zaltoprofen. 

Model simulations in this study were performed only for the maximum or minimum change in covariates in healthy adults. In the future, the model can be expanded by obtaining clinical data of zaltoprofen in patients with abnormal covariate levels (such as renal or hepatic impairment). As implied in Figure 4 and Figure 5, the dose of zaltoprofen was adjusted according to the degree of variation in the effective covariates, CrCL and albumin. That is, when CrCL and albumin levels were 80 mL/min and 5.5 g/dL, respectively, the mean plasma concentration of zaltoprofen in the steady-state was increased by approximately 1.49 times compared to the normal group, and the continuation of this state was expected to be associated with increased side effects. When CrCL and albumin levels were 130 mL/min and 3.5 g/dL, respectively, the mean plasma concentration of zaltoprofen in the steady-state was approximately 0.43 times lower than that of the normal group, and the persistence of this state was expected to be associated with treatment failure. Therefore, the appropriate increase or decrease of zaltoprofen dose according to CrCL and albumin levels is important when maintaining optimal drug concentrations and reducing side effects. Reducing (when CrCL and albumin levels are 80 mL/min and 5.5 g/dL, respectively) or increasing (when CrCL and albumin levels are 130 mL/min and 3.5 g/dL, respectively) the drug in each patient group may achieve the same level of zaltoprofen plasma concentration as patients with general stable biochemical markers while maintaining a dosing interval of 24 h or 8 h. 

Considering pharmacokinetics, dose adjustment was suggested based on the general assumption that drug efficacy and side effects would be proportional to the degree of plasma exposure. In the future, it may be necessary to further adjust doses considering the pharmacodynamics of zaltoprofen. To this end, clinical pharmacodynamic studies of zaltoprofen are warranted, along with finding useful biomarkers (such as the degree of COX inhibition). This model simulation of zaltoprofen was a novel scientific quantitative approach to reasonably predict pharmacokinetic profiles when it is difficult to do so for a large number of subjects with various CYP2C9 genetic polymorphisms and biochemical values. Here, we provide useful scientific data for the efficient and precise clinical application of zaltoprofen.

## 4. Materials and Methods

### 4.1. Study Workflow

This study was conducted in three steps. First, we performed population modeling and validation based on zaltoprofen pharmacokinetic data [19]. The pharmacokinetic data used for modeling were derived from previously reported bioequivalence results [19]. Second, we externally validated the developed model using previously reported pharmacokinetic results [25,26,27] according to zaltoprofen exposure. The graphical data used for external validation was semi-automatically read and digitized using WebPlotDigitizer software (4.5 version). Third, we predicted the pharmacokinetics according to random numerical fluctuations of effective covariates in the established model and adjusted the dosage accordingly through model simulation.

### 4.2. Population Pharmacokinetic Modeling

Pharmacokinetic results obtained from a bioequivalence study of 80 mg of zaltoprofen in 26 healthy Korean males were used to develop this PPK model. As suggested in a previous report [19], the clinical trial was thoroughly reviewed and officially approved (by the Institutional Review Board of the Institute of Bioequivalence and Bridging Study, Chonnam National University, Gwangju, Republic of Korea; Approval No. 060118; 11.25.2005). Model construction and analysis were performed with a nonlinear mixed effects approach using Phoenix NLME (8.3 version, Pharsight, Certara Inc., NJ, USA) software. The PPK parameters were estimated via the first-order conditional estimates method with extended least squares estimation (with *ŋ*–*ε* interaction). Model development involved two major steps, the first of which was establishing a basic structural model to explain the plasma concentration of zaltoprofen following oral administration. This included determining the number of compartments, whether the lag time was reflected in zaltoprofen oral absorption or the mechanistic structuring of the absorption process, and error models to account for residual and interindividual variabilities (IIVs). The second step involved the search for effective covariates in explaining the individual pharmacokinetic variability of zaltoprofen. Biochemical factors and genetic polymorphisms of CYP2C9 were considered as potential effective covariates. Analyses of biochemical factors and CYP2C9 genotypes were performed on subjects participating in the bioequivalence test. The methods are presented in the Supplementary Materials (SM; Subsections 1 and 2). Suitable models for each step were selected using various statistical significance tools derived via the Phoenix NLME. These included Akaike’s information criterion (AIC), -2LL, and GOF plots, and the significance levels according to the total number of parameters applied to the model (increasing or decreasing degrees of freedom). Significance was determined based on a *p*-value of 0.05 in chi-square distribution.

The potential covariates, CYP2C9 genetic polymorphism information and biochemical parameters, were sequentially applied to the IIV model of the pharmacokinetic parameters used in the model as categorical and continuous data, respectively. In the case of biochemical parameters, pharmacokinetic parameters for each individual were calculated by NCA beforehand, and correlations between the values and biochemical factors were screened through linear regression analysis. Methods for calculating pharmacokinetic parameters via NCA are presented in SM (Subsection 3). As a result of screening, biochemical covariates with correlations of about 30% or more were selected as possible candidate covariates for the final application and reflected in the IIV model factor. Both forward addition and backward elimination were considered in the application of the covariates, and *p*-values of 0.05 and 0.01 in chi-square distribution were set as the significance criterion, respectively.

### 4.3. Model Validation

A PPK model of zaltoprofen was fully evaluated and validated visually or numerically using Phoenix NLME and R-software (4.2.2 version, R-Core Team). The model evaluation was performed using widely used population-scale model validation tools. These included GOF (including distribution of residuals), VPC, bootstrapping, and NPDE. The approaches to each model validation tool are presented in SM (Subsection 4).

### 4.4. External Model Validation

Previous clinical pharmacokinetic results for zaltoprofen [25,26,27] were applied to externally validate the zaltoprofen PPK model. All were population mean plasma concentration profiles performed on healthy adults. Doses of 80, 160, and 240 mg were used for the single exposures while 80 mg was used for multiple exposures. Information on past clinical studies applied to external validation is presented in Table S6. The VPC approach was mainly applied to check whether past reported results for each dose group were well-included in the predicted areas of the zaltoprofen PPK model established in this study (according to the dose change). We confirmed through the VPC approach whether the results of multiple-dose observations were also well-included in the prediction ranges of the model.

### 4.5. Model Simulation

To proceed with the simulation based on the zaltoprofen PPK model, the final established and validated zaltoprofen PPK model structure was fixed. The parameter values were fixed as the common mean values of the group, except where covariate reflection was considered. Model simulations were performed by predicting and comparing the changes in the pharmacokinetic parameter values according to the numerical changes of the covariates reflected in the final model. Model simulations were possible for different exposure scenarios, such as single and multiple exposures with varying doses of zaltoprofen or varying dose intervals. Model simulations were performed using the Simulation and Prediction engines of Phoenix NLME. The predictive profiles of pharmacokinetics between groups at multiple zaltoprofen exposures according to the covariate changes were compared with the 50% equivalent, or the approximate mean of each group. Differences were quantitatively confirmed by comparing the steady-state plasma zaltoprofen concentrations between the groups, with dose adjustments and model simulation based on these results.

## 5. Conclusions

In this study, a clinically applicable PPK model of zaltoprofen was reported considering CrCL and albumin levels. The established PPK model quantified the difference in pharmacokinetics according to changes in CrCL and albumin levels and zaltoprofen dosage using a simulation tool. This study confirms that CrCL and albumin variability are the most important factors in individual zaltoprofen pharmacokinetic variability, which must be considered in a dosage regimen. Increases and decreases in CrCL and albumin levels cause significant changes in plasma exposure to zaltoprofen, and pharmacokinetic-based dose adjustments are needed for effective drug therapy, assuming a proportional relationship between plasma exposure and efficacy. Although the variability of zaltoprofen pharmacokinetics according to CYP2C9 genetic polymorphisms requires further study, this study shows that the pharmacokinetic fluctuations caused by *3 allele heterozygotes were not significant and did require consideration for dose adjustment in clinical practice. This study presents a very important starting point for individualized zaltoprofen drug therapy and precision medicine.

## Data Availability

All data and related materials are accessible in this manuscript and Supplementary Materials.

## Supplementary Materials

The following supporting information can be downloaded at: https://www.mdpi.com/article/10.3390/ph16020161/s1, Figure S1: Comparison graphs of zaltoprofen pharmacokinetic parameters between CYP2C9*1/*1 and *1/*3; Figure S2: Pharmacokinetic profiles according to CYP2C9 genotypes (CYP2C9*1/*1 and *1/*3) and means in 26 Korean males following single oral administration of 80 mg zaltoprofen; Figure S3: Relationship between subjects’ physicochemical parameters and individual predicted pharmacokinetic parameters; Figure S4: Relationship between subjects’ physicochemical parameters and individual predicted pharmacokinetic parameters; Figure S5: Goodness-of-fit plots of final pharmacokinetic model for zaltoprofen; Figure S6: Normalized prediction distribution error plots for the population pharmacokinetic model of zaltoprofen; Figure S7: VPC of the final pharmacokinetics model for zaltoprofen; Figure S8: VPC of pharmacokinetics of the final model for zaltoprofen with stratification according to CYP2C9 genotypes; Table S1: The basic pharmacokinetic structural model of zaltoprofen; Table S2: Stepwise selection of potential covariates in the population pharmacokinetic model of zaltoprofen; Table S3: Parameter values of the final population pharmacokinetic model for zaltoprofen; Table S4: Bootstrap results of the final population pharmacokinetic model for zaltoprofen; Table S5: Demographic information of the healthy Korean males on single oral administration of 80 mg zaltoprofen (*n* = 26); Table S6: Past clinical studies applied to the external validation.
