# Supplementary material for: Population Pharmacokinetic Modeling of Zaltoprofen in Healthy Adults: Exploring the Dosage Regimen
Source: Pharmaceuticals (Basel). 2023 Jan 22;16(2):161. doi: 10.3390/ph16020161 (PMC9962663; doi:10.3390/ph16020161)
Supplement: Supplementary file 1 [file pharmaceuticals-16-00161-s001.zip › pharmaceuticals-2136670-supplementary.pdf]

Population pharmacokinetic modeling of zaltoprofen in healthy adults: Exploring the dosage regimen

Supplementary Materials

Ji-Hun Jang[1,†], Seung-Hyun Jeong[2,†], and Yong-Bok Lee[1,*]

[1] College of Pharmacy, Chonnam National University, 77 Yongbong-ro, Buk-gu, Gwangju, 61186, Republic of Korea
[2] College of Pharmacy, Sunchon National University, 255 Jungang-ro, Suncheon-si, Jeollanam-do, 57922, Republic of Korea

[†] These authors (Seung-Hyun Jeong & Ji-Hun Jang) contributed equally as first-authors to this manuscript.

*Corresponding author:
Yong Bok Lee
College of Pharmacy, Chonnam National University, 77 Yongbong-ro, Buk-gu, Gwangju, 61186, Republic of Korea
Tel.: +82-62-530-2931; Fax: +82-62-530-0106
E-mail: leeyb@chonnam.ac.kr

Supplementary Materials

1. Determination of clinical biochemistry parameters

Blank serum samples obtained immediately before oral zaltoprofen administration (0 h) were used to analyze the clinical biochemical parameters, including total proteins, albumin, alkaline phosphatase (ALP), aspartate transaminase (AST), alanine transaminase (ALT), total bilirubin, blood urea nitrogen, creatinine, creatinine clearance (CrCL), and glomerular filtration rate (GFR). Determination of clinical biochemical parameter values was performed by serological analysis through a dry automated analyzer. The analytical instrument used was a microsides VITROS (Ortho Clinical Diagnostics, NJ, USA) operated by reflectance spectrophotometry. CrCL was calculated based on the Cockcroft-Gault equation: [(140 - age) × body weight (kg)/serum creatinine (mg/dL) × 72]. GFR was calculated using the Modification-of-Diet-in-Renal-Disease formula: 186.3 × [serum creatinine $(mg/dL)^{-1.154}$ × age $(year)^{-0.203}$]. Body mass index (BMI) and body surface area (BSA) were calculated based on the individuals' weight and height information. BMI was calculated based on the Kaup index: [body weight (kg)/$height^2$ (m^2)]. BSA was calculated based on the Mosteller formula: $\sqrt{(\text{height (cm)} \times \text{weight (kg)}/3600)}$.

2. Determination of *CYP2C9*1 and *3* alleles

Only the alleles of *1 and *3 were confirmed after analyzing the CYP2C9 genotypes. The genotypes of each individual at the CYP2C9*3 allele were determined using polymerase chain reaction-restriction fragment length polymorphism (PCR-RFLP) to amplify the portions of exon 7 containing A1075→C SNP as previously described, with minor modifications. For the CYP2C9*3 allele genotype, PCR was carried out in a 20 μL reaction mixture containing PCR PreMix, 200–300 ng of genomic DNA, and 10 pmol of each primer. The sequences of the forward and reverse primers were 5′-TGCACGAGGTCCAGAGGTAC-3′ and 5′-ACAAACTTACCTTGGGAATGAGA-3′ for the CYP2C9*3 genotype.

PCR was conducted by an initial denaturation at 94 °C for five min followed by 30 cycles, each consisting of denaturation at 94 °C for one min, annealing at 55 °C for one min, and

extension at 72 °C for one min. The final extension was performed at 72 °C for 10 min. The amplified 105-bp fragment consisted of 10 bp of intron and 95 bp of exon 7. PCR products were electrophoresed on 2% agarose gel and visualized by ultraviolet transillumination after staining with ethidium bromide. Aliquots of each PCR product were digested with restriction enzyme *KpnI* for CYP2C9*3 at 37 °C for 1.5 h. The digested fragments were separated by electrophoresis using a 2.5% agarose gel and visualized under ultraviolet light after staining the gel with ethidium bromide. A cross-platform confirmation procedure was carried out through direct sequencing of 5–10% of all samples using real-time PCR to reproduce the genotyping method (PCR-RFLP). Sequencing was performed by dideoxy chain termination using BigDye technology (Applied Biosystems, Foster City, CA, USA). PCR products were purified using a magnesium-chloride/ethanol-based protocol and run on an ABI 3100 Genetic Analyzer (AB Applied Biosystems). The genotype concordance rate in all samples was 100%.

3. Non-compartment analysis

Calculations of the basic pharmacokinetic parameters of zaltoprofen were performed by non-compartment analysis (NCA) using Phoenix WinNonlin software (8.3 version, Pharsight, Certara Inc.). The area under the curve from 0 h to infinite (AUC_{inf}) was calculated as the sum of $AUC_{0\text{–}t}$ and C_{last}/k, where C_{last} is the final measured concentration, t is the time in C_{last}, and k is the elimination rate constant at terminal phase. $AUC_{0\text{–}t}$ was calculated using a linear trapezoidal rule from 0 to t h after oral administration of zaltoprofen. The half-life ($T_{1/2}$) was calculated as 0.693/k, and the volume of the distribution (V/F) was calculated as dose/k · $AUC_{0\text{–}\infty}$. The clearance (CL/F) was calculated by dividing the dose of zaltoprofen by $AUC_{0\text{–}\infty}$, where F is the oral administration bioavailability. The peak plasma drug concentration (C_{max}) and time to reach C_{max} (T_{max}) were determined from the plasma zaltoprofen concentration-time curves of each individual.

4. Model qualification tools

The Goodness-of-fit (GOF) was confirmed using diagnostic scatter plots as follows: (A) population-predicted concentrations (PRED) versus observed concentrations (DV), (B)

individual-predicted concentrations (IPRED) versus DV, (C) PRED versus conditional weighted residuals (CWRES), (D) time after dose (IVAR) versus CWRES, (E) quantile-quantile (QQ) plot of the components of CWRES, and (F) QQ plot of the components of individual weighted residuals (IWRES). A visual predictive check (VPC) of the final established model was performed using the VPC option of Phoenix NLME (version 8.3). There were 100 simulations for the VPC. The IVAR–DV concentration data were graphically superimposed on the median values and the 5th and 95th percentiles of the IVAR-simulated concentration profiles. If the DV concentration data were approximately distributed within the 95th and 5th prediction interval, the model was expected to be precise. The stability of the final model was confirmed using non-parametric bootstrap analysis via the bootstrap option of Phoenix NLME. A total of 1,000 replicates were generated by repeated random sampling with replacement from the original dataset. The estimated parameter values, such as the standard errors [SE; including confidence interval (CI)] and medians from the bootstrap procedure, were compared with those estimated from the original dataset. The normalized prediction distribution error (NPDE) was used to evaluate the predictive performance of the model based on a Monte Carlo simulation with the R-package. NPDE results were summarized graphically using (A) quantile–quantile plot of the NPDE, (B) a histogram of the NPDE, (C) a scatterplot of NPDE versus IVAR, and (D) a scatterplot of NPDE versus PRED. The NPDE will follow a normal distribution if the predictive performance is satisfied (Shapiro–Wilk test) with a mean value of zero (*t*-test) and a variance of one (Fisher's test).

Table S1. The basic pharmacokinetic structural model of zaltoprofen.

Model	Description	nParameter [a]	-2LL	AIC	△-2LL	△AIC	Compared with
**Compartment model**							
01	1-compartment	7	2854.25	2876.53	-	-	-
02*	2-compartment	11	643.72	665.72	-2210.527884	-2210.811473	01
03	3-compartment	15	643.59	665.68	-0.13	-0.04	02
**Absorption model**							
02*	No lag-time	11	643.72	665.72	-	-	-
02-01	Add lag-time	13	714.30	726.57	70.5819	60.8527	02
02-02	No lag-time; Non sequential two absorption compartment**	15	644.30	666.73	0.5819	1.0148	02
02-03	No lag-time; Sequential two absorption compartment**	13	652.53	665.19	8.8152	-0.5253	02

**Residual error model**							
02	Additive	11	643.72	665.72	-	-	-
02-04*	Proportional	11	168.75	190.75	-474.97	-474.97	02
02-05	Log additive	11	443.37	465.37	-200.35	-200.35	02
02-06	Mixed	12	168.77	192.77	-474.95	-472.95	02
02-07	Power	11	643.72	665.72	0.00	0.00	02
**Inter-individual variability (IIV) model**							
02-04-01	Remove IIV [b] K_a	10	227.00	247.00	58.25	56.25	02-04
02-04-02	Remove IIV [b] V/F	10	168.74	188.74	-0.01	-2.01	02-04
02-04-03	Remove IIV [b] V_2/F	10	181.31	201.31	12.56	10.56	02-04
02-04-04	Remove IIV [b] CL/F	10	247.87	267.87	79.13	77.13	02-04
02-04-05	Remove IIV [b] CL_2/F	10	171.34	191.34	2.60	0.60	02-04
02-04-06*	Remove IIV [b] V/F, CL_2/F	9	168.74	186.74	0.000	-2.000	02-04-02

[a] nParameter means the total number of parameters applied to the model.

[b] IIV means the inter-individual variation.

* means the model selected in each step.

** means a kind of multiple absorption compartment models.

Table S2. Stepwise selection of potential covariates in the population pharmacokinetic model of zaltoprofen.

Model	Description	OFV	△OFV	Compared with	nParameter [a]
1	Base model	168.74	-	-	9
2	Genotype** on K_a	167.19	-1.544	Base model	10
3	Genotype** on V_2/F	165.01	-3.722	Base model	10
4	Genotype** on CL/F	164.89	-3.845	Base model	10
5	CrCL on V_2/F	168.74	0.000	Base model	10
6	CrCL on CL/F	160.73	-8.002	Base model	10
7	BSA on CL/F	166.66	-2.076	Base model	10
8	Albumin on CL/F	161.72	-7.013	Base model	10
9	Genotype** and CrCL on CL/F	160.36	-0.374	Model 6	11
10*	CrCL and albumin on CL/F	153.18	-7.552	Model 6	11
11	Genotype** and CrCL and albumin on CL/F	153.23	0.046	Model 10	12

[a] nParameter is the total number of parameters applied to the model.

* indicates the final model.

** indicates genetic polymorphisms of the CYP2C9 genotypes *1/*1 and *1/*3.

Table S3. Parameter values of the final population pharmacokinetic model for zaltoprofen.

Parameter	Estimate	SE [a]	RSE (%) [a]	Shrinkage (%)	IIV [b] (%)
tv K_a (1/h)	1.73	0.15	8.56		
tv V/F (L)	4.88	1.28	26.32		
tv CL/F (L/h)	43.70	2.12	4.85		
tv V_2/F (L)	40.56	7.02	17.32		
tv CL_2/F (L/h)	5.61	0.63	11.16		
dCL/FdCrCL	0.48	0.17	35.09		
dCL/FdAlbumin	-1.83	0.64	35.18		
ω^2_{Ka}	0.17	0.06	33.95	0.09	40.63
$\omega^2_{V2/F}$	0.24	0.08	32.31	0.21	48.55
$\omega^2_{CL/F}$	0.01	0.00	46.60	0.34	9.80
ε	0.38	0.02	6.03		

[a] SE and RSE indicate standard error and relative standard error, respectively.

[b] IIV indicates the inter-individual variation.

tv indicates the typical value.

Table S4. Bootstrap results of the final population pharmacokinetic model for zaltoprofen.

Parameter	Final model		Bootstrap (*n* = 1000)	
	Estimate	95% CI [a]	Median	95% CI [a]
tv K_a (1/h)	1.73	1.43 - 2.02	1.73	1.52 - 1.96
tv V/F (L)	4.88	2.35 - 7.41	4.59	2.98 - 8.18
tv CL/F (L/h)	43.70	39.52 - 47.87	43.32	38.36 - 48.01
tv V_2/F (L)	40.56	26.74 - 52.39	40.69	29.45 - 58.85
tv CL_2/F (L/h)	5.61	4.38 - 6.84	5.52	4.51 - 6.66
dCL/FdCrCL	0.48	0.15 - 0.81	0.48	0.16 - 0.81
dCL/FdAlbumin	-1.83	-3.10 - -0.56	-1.70	-3.11 - -0.80
ω^2_{Ka}	0.17	0.06 - 0.28	0.15	0.07 - 0.24
$\omega^2_{V2/F}$	0.24	0.09 - 0.39	0.21	0.06 - 0.37
$\omega^2_{CL/F}$	0.01	0.00 - 0.02	0.01	0.00 - 0.02
ε	0.38	0.34 - 0.43	0.38	0.34 - 0.42

[a] CI indicates confidence interval.

tv indicates typical value.

Table S5. Demographic information of the healthy Korean males on single oral administration of 80 mg zaltoprofen ($n = 26$).

**Demographic**	**Unit**	**Value (mean ± standard deviation)**
Age	Year	23.19 ± 2.26
Weight	kg	64.73 ± 8.08
Height	cm	172.64 ± 5.95
Total proteins	g/dL	7.35 ± 0.38
Albumin	g/dL	4.92 ± 0.18
ALP	IU/L	73.23 ± 15.30
AST	IU/L	19.23 ± 3.86
ALT	IU/L	18.23 ± 8.28
Total bilirubin	mg/dL	1.03 ± 0.41
Blood urea nitrogen	mg/dL	12.93 ± 2.29
Creatinine	mg/dL	0.98 ± 0.09

CrCL	mL/min	107.53 ± 17.28
BMI	kg/m^2	21.74 ± 2.60
BSA	m^2	1.76 ± 0.12
GFR	mL/min	101.34 ± 11.53

ALP, alkaline phosphatase; AST, aspartate transaminase; ALT, alanine transaminase; CrCL, creatinine clearance; BMI, body mass index; BSA, body surface area; GFR, glomerular filtration rate.

Table S6. Past clinical studies applied to the external validation.

References	Administration	Group	Sampling time
Kang et al. (2006) [1]	80 mg/single oral dose	26 healthy Korean males	0-24 h (after dose)
Lee et al. (2006) [2]	160 mg/single oral dose	24 healthy Korean males	0-24 h (after dose)
Li et al. (2011) [3]	80, 160, 240 mg/single oral dose	12 healthy Chinese individuals	0-24 h (after dose)
	80 mg/multiple oral doses (for six days at eight h intervals; one dose on the seventh day after administration)	12 healthy Chinese individuals	120 h, 144 h, and 168-192 h (after first dose)

Supplementary figure captions

Figure S1. Comparison graphs of zaltoprofen pharmacokinetic parameters [(A) $T_{1/2}$, (B) T_{max}, (C) C_{max}, (D) AUC_{0-t}, (E) AUC_{inf}, (F) V/F, and (G) CL/F] between CYP2C9*1/*1 and *1/*3. * reveals a statistically significant difference between CYP2C9*1/*1 and *1/*3 with $p < 0.05$. The p-values for (A), (B), (C), (D), (E), (F), and (G) were 0.053, 0.024, 0.925, 0.185, 0.078, 0.287, and 0.093, respectively. Significance was confirmed through the *t*-test after checking variance equality (A, C, D, E, F, and G) between groups by the *F*-test. If equal variances were not established (B), a *t*-test based on the assumption of heterogeneity was performed.

Figure S2. Pharmacokinetic profiles according to CYP2C9 genotypes (CYP2C9*1/*1 and *1/*3) and means in 26 Korean males following single oral administration of 80 mg zaltoprofen. Vertical bars indicate the standard deviation.

Figure S3. Relationship between subjects' physicochemical parameters and individual predicted pharmacokinetic parameters. (A) CL/F of zaltoprofen according to CrCL, (B) AUC_{0-t} of zaltoprofen according to CrCL, (C) CL/F of zaltoprofen according to albumin, (D) CL/F of zaltoprofen according to GFR, and (E) CL/F of zaltoprofen according to BSA.

Figure S4. Relationship between subjects' physicochemical parameters and individual predicted pharmacokinetic parameters. (A) CL/F of zaltoprofen according to ALT, (B) CL/F of zaltoprofen according to ALP, (C) CL/F of zaltoprofen according to BUN, (D) CL/F of zaltoprofen according to BMI, (E) V/F of zaltoprofen according to BSA, (F) V/F of zaltoprofen according to BMI, and (G) V/F of zaltoprofen according to albumin.

Figure S5. Goodness-of-fit plots of final pharmacokinetic model for zaltoprofen. (A) Population-predicted concentrations (PRED) against observed plasma concentration (DV), (B) individual-predicted concentrations (IPRED) against DV, (C) PRED against conditional weighted residuals (CWRES), (D) time after dose (IVAR) against CWRES, (E) quantile–quantile (QQ) plot of CWRES components, and (F) QQ plot of individual weighted residual (IWRES) components.

Figure S6. Normalized prediction distribution error (NPDE) plots for the population

pharmacokinetic model of zaltoprofen. (A) Quantile-quantile plots of NPDE versus the theoretical N (0, 1) distribution. (B) Histogram of the NPDE distribution overlaid with the standard Gaussian distribution density. (C) Scatter plot of time versus NPDE. (D) Scatterplot of predictions versus NPDE.

Figure S7. VPC of the final pharmacokinetics model for zaltoprofen. Observed concentrations (after oral administration of 80 mg) are depicted by the black dots. The 95th, 50th, and 5th percentiles of the predicted concentrations are represented by the black dashed lines. The 95% confidence intervals (CI) for the predicted 5th and 95th percentiles are represented by the blue shaded regions. The 95% CI for the predicted 50th percentiles are represented by the red shaded regions. X-axis indicates the time after oral zaltoprofen administration and the Y-axis shows the observed and predicted plasma concentrations of zaltoprofen.

Figure S8. VPC of pharmacokinetics of the final model for zaltoprofen [with stratification according to CYP2C9 genotypes; (A) CYP2C9*1/*1 and (B) CYP2C9*1/*3]. Observed concentrations (after 80 mg oral administration) are depicted by the black dots. The 95th, 50th, and 5th percentiles of the predicted concentrations are represented by the black dashed lines. The 95% confidence intervals (CI) for the predicted 5th and 95th percentiles are represented by the blue shaded regions. The 95% CI for the predicted 50th percentiles are represented by the red shaded regions. X-axis indicates the time after oral zaltoprofen administration and the Y-axis shows the observed and predicted plasma concentrations of zaltoprofen.

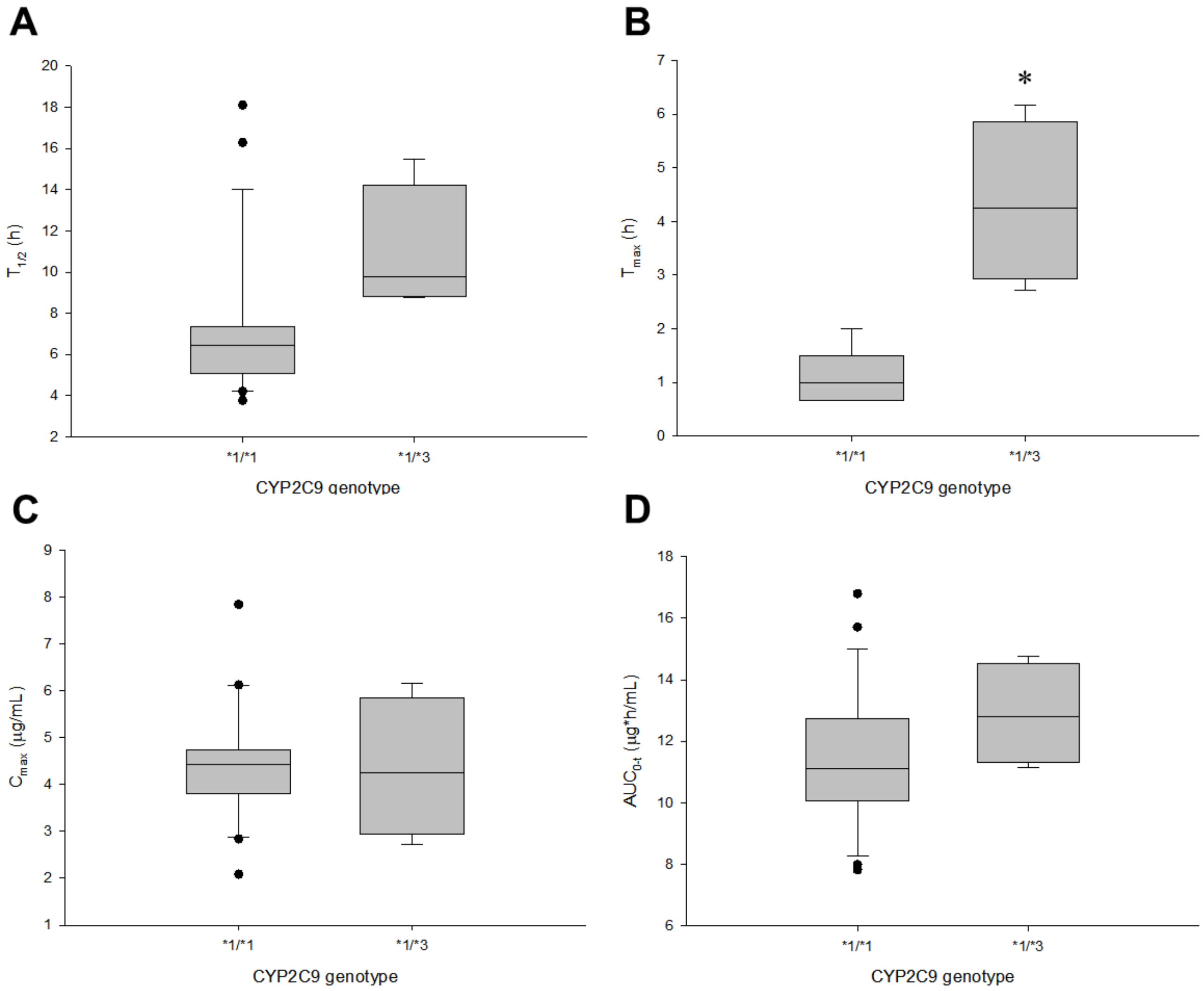

A
20
18
16
14
12
10
8
6
4
2
T1/2 (h)
*1/*1
*1/*3
CYP2C9 genotype
B
7
6
5
4
3
2
1
0
Tmax (h)
*
*1/*1
*1/*3
CYP2C9 genotype
C
9
8
7
6
5
4
3
2
1
Cmax (µg/mL)
*1/*1
*1/*3
CYP2C9 genotype
D
18
16
14
12
10
8
6
AUC0-t (µg*h/mL)
*1/*1
*1/*3
CYP2C9 genotype

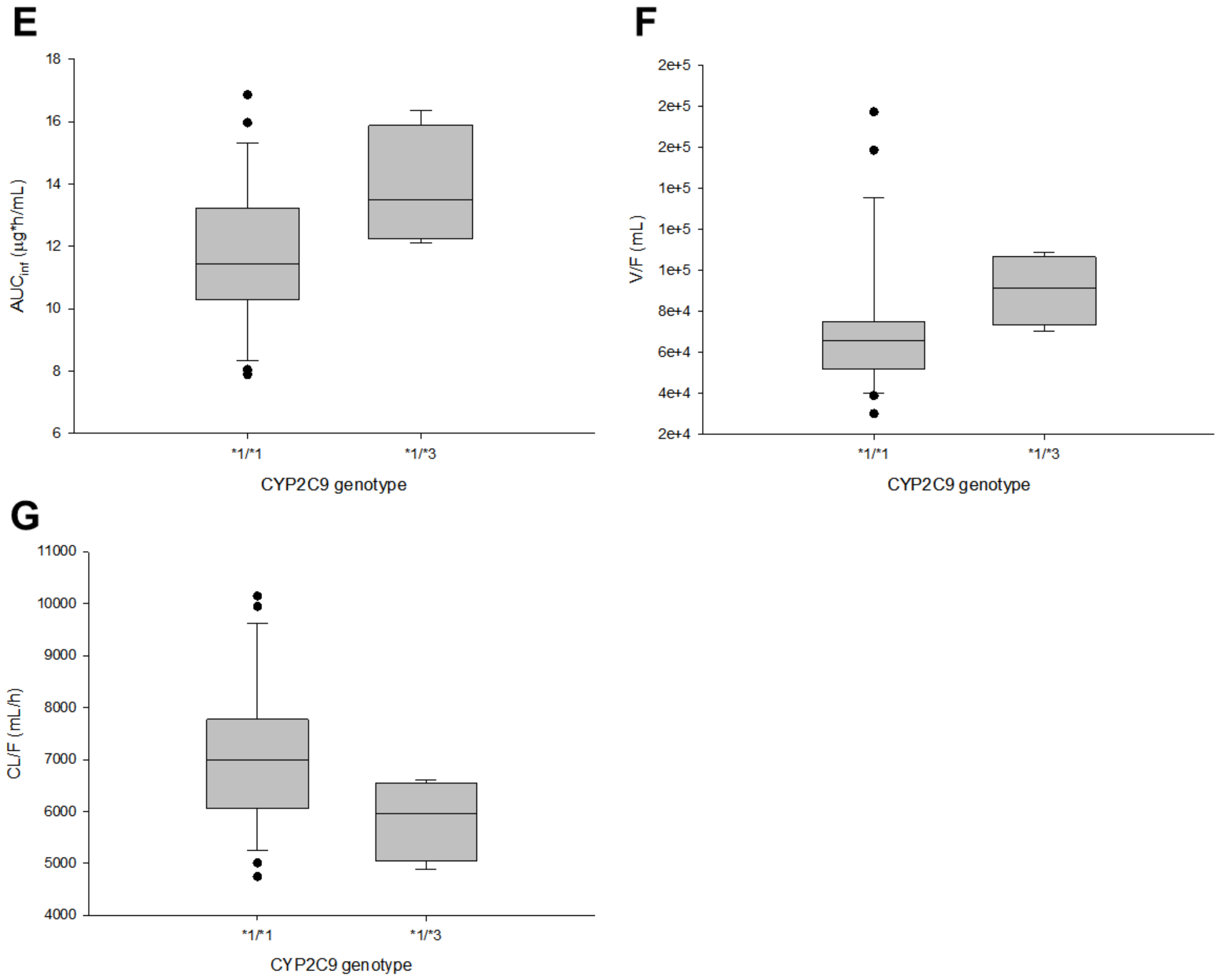
E
AUC$_{inf}$ (µg*h/mL)
18
16
14
12
10
8
6
*1/*1
*1/*3
CYP2C9 genotype
F
V/F (mL)
2e+5
2e+5
2e+5
1e+5
1e+5
1e+5
8e+4
6e+4
4e+4
2e+4
*1/*1
*1/*3
CYP2C9 genotype
G
CL/F (mL/h)
11000
10000
9000
8000
7000
6000
5000
4000
*1/*1
*1/*3
CYP2C9 genotype

Figure S1

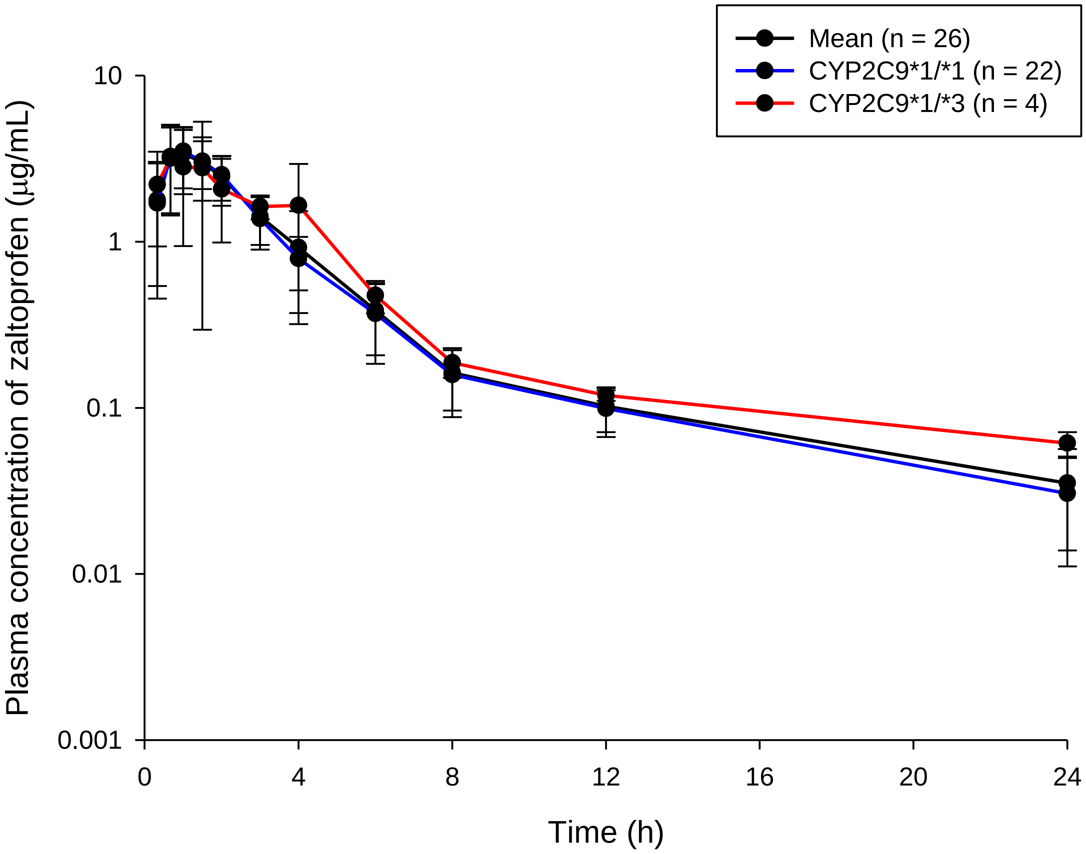
Mean (n = 26)
CYP2C9*1/*1 (n = 22)
CYP2C9*1/*3 (n = 4)
Plasma concentration of zaltoprofen (μg/mL)
10
1
0.1
0.01
0.001
0
4
8
12
16
20
24
Time (h)

Figure S2

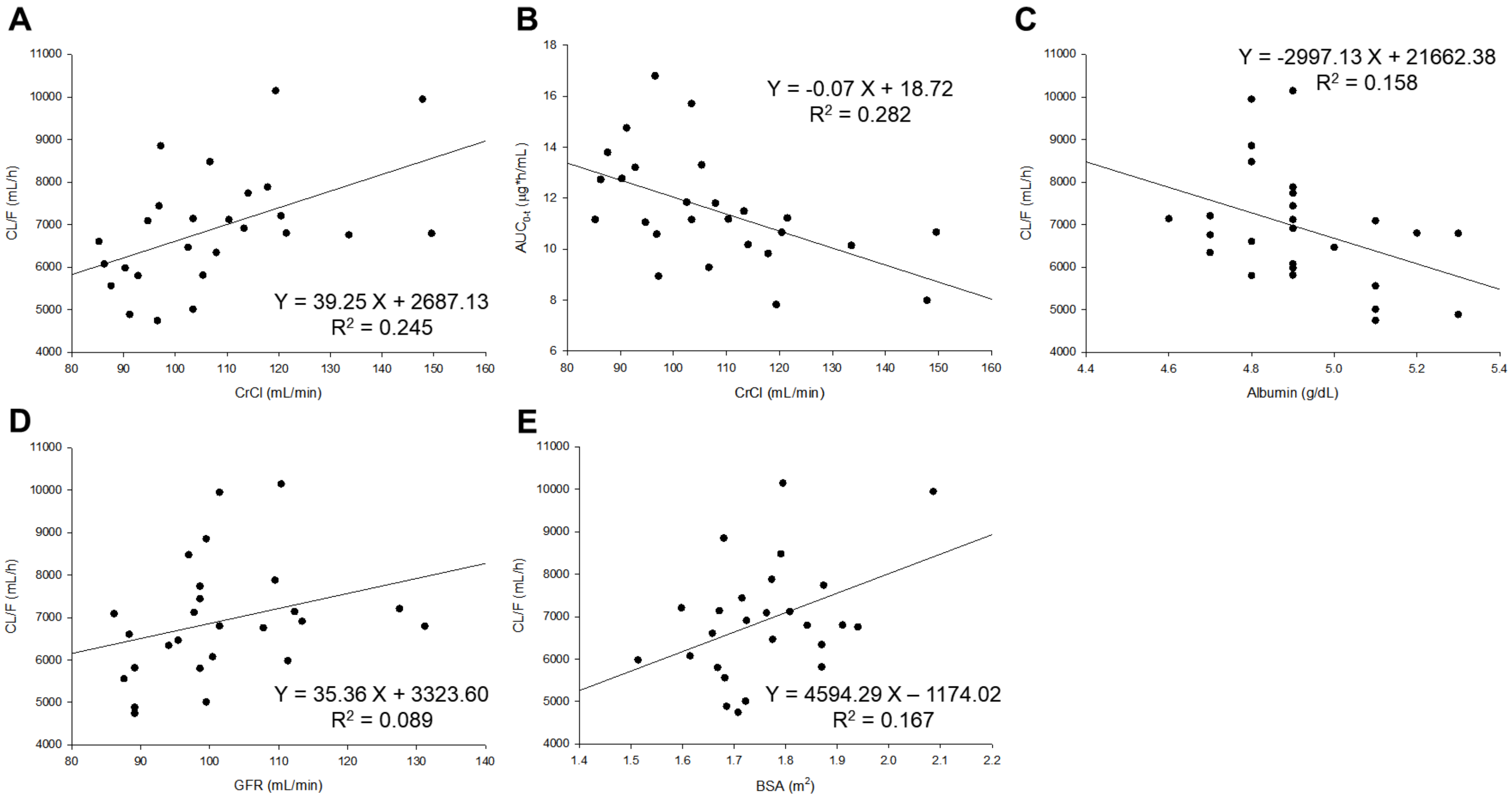
A
CL/F (mL/h)
CrCl (mL/min)
Y = 39.25 X + 2687.13
R2 = 0.245
B
AUC0-t (µg*h/mL)
CrCl (mL/min)
Y = -0.07 X + 18.72
R2 = 0.282
C
CL/F (mL/h)
Albumin (g/dL)
Y = -2997.13 X + 21662.38
R2 = 0.158
D
CL/F (mL/h)
GFR (mL/min)
Y = 35.36 X + 3323.60
R2 = 0.089
E
CL/F (mL/h)
BSA (m2)
Y = 4594.29 X – 1174.02
R2 = 0.167

Figure S3

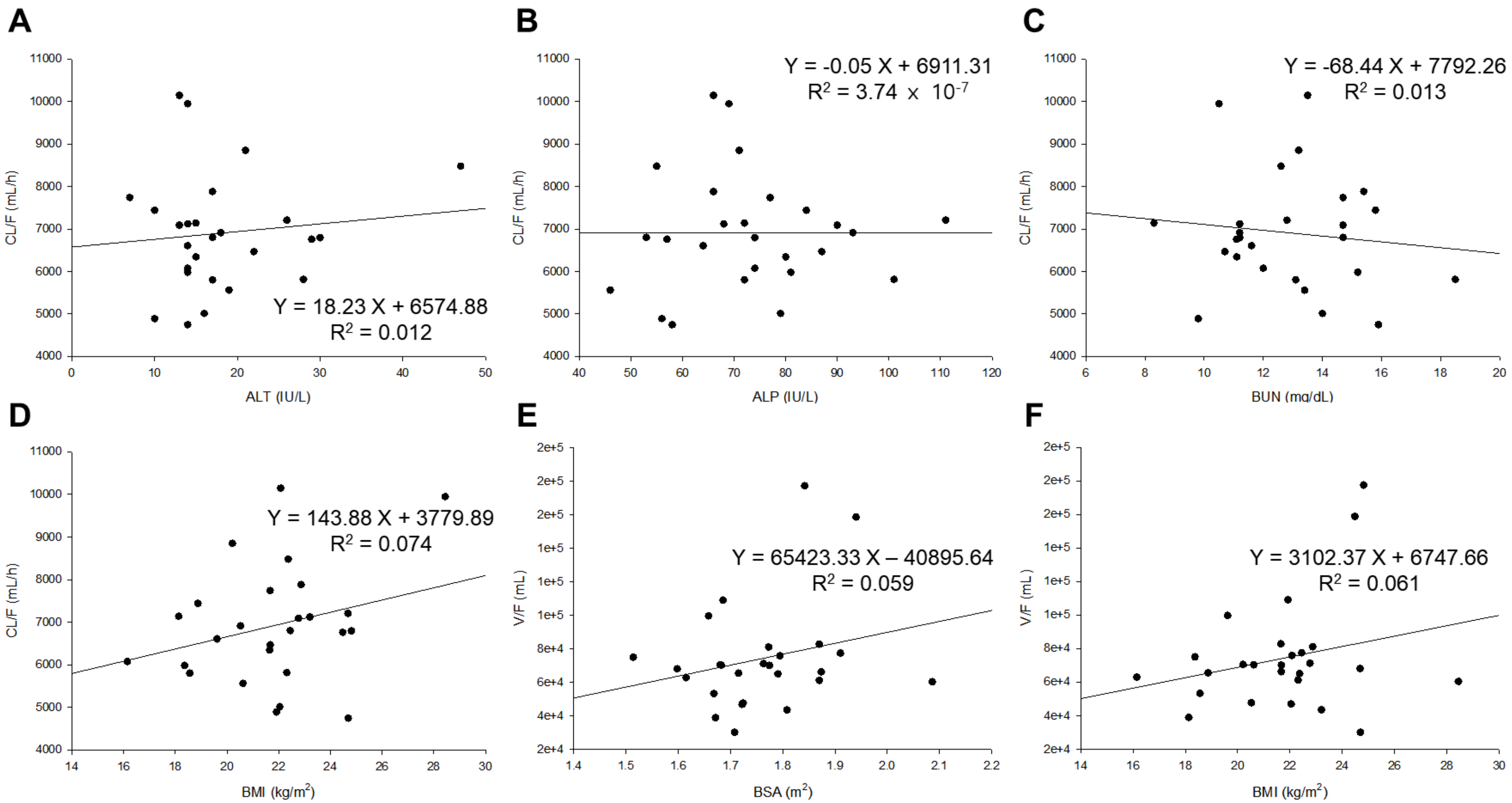
A
CL/F (mL/h)
ALT (IU/L)
Y = 18.23 X + 6574.88
R2 = 0.012
B
CL/F (mL/h)
ALP (IU/L)
Y = -0.05 X + 6911.31
R2 = 3.74 x 10-7
C
CL/F (mL/h)
BUN (mg/dL)
Y = -68.44 X + 7792.26
R2 = 0.013
D
CL/F (mL/h)
BMI (kg/m2)
Y = 143.88 X + 3779.89
R2 = 0.074
E
V/F (mL)
BSA (m2)
Y = 65423.33 X – 40895.64
R2 = 0.059
F
V/F (mL)
BMI (kg/m2)
Y = 3102.37 X + 6747.66
R2 = 0.061

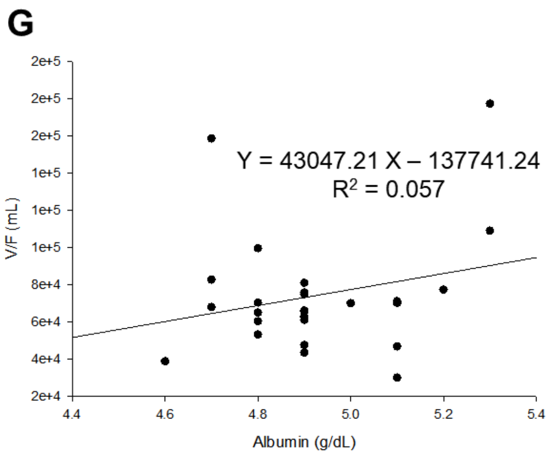
G
Y = 43047.21 X – 137741.24
R2 = 0.057
V/F (mL)
2e+5
2e+5
2e+5
1e+5
1e+5
1e+5
8e+4
6e+4
4e+4
2e+4
4.4
4.6
4.8
5.0
5.2
5.4
Albumin (g/dL)

Figure S4

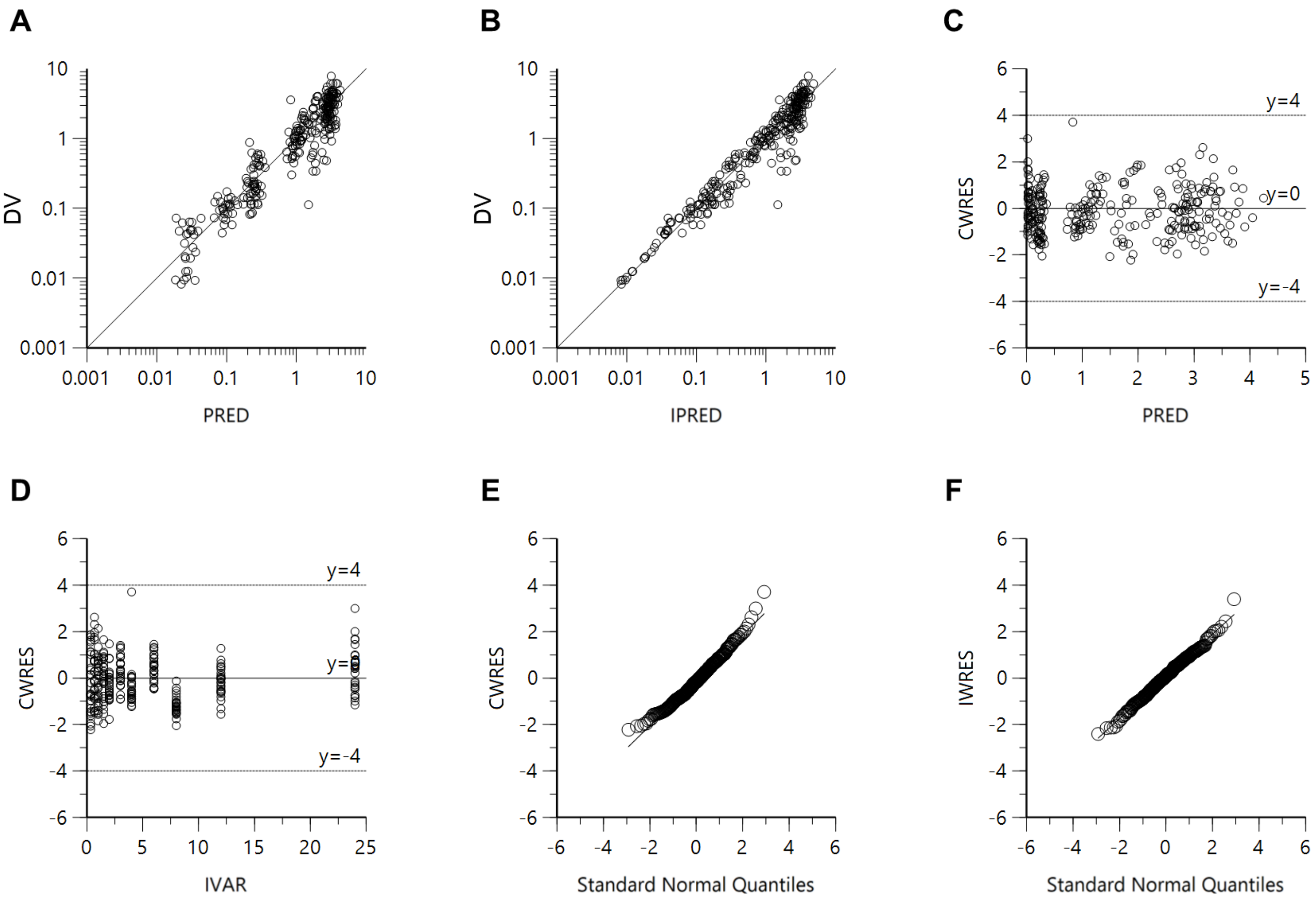
A
B
C
D
E
F
DV
PRED
IPRED
CWRES
IWRES
IVAR
Standard Normal Quantiles
y=4
y=0
y=-4
0.001
0.01
0.1
1
10

Figure S5

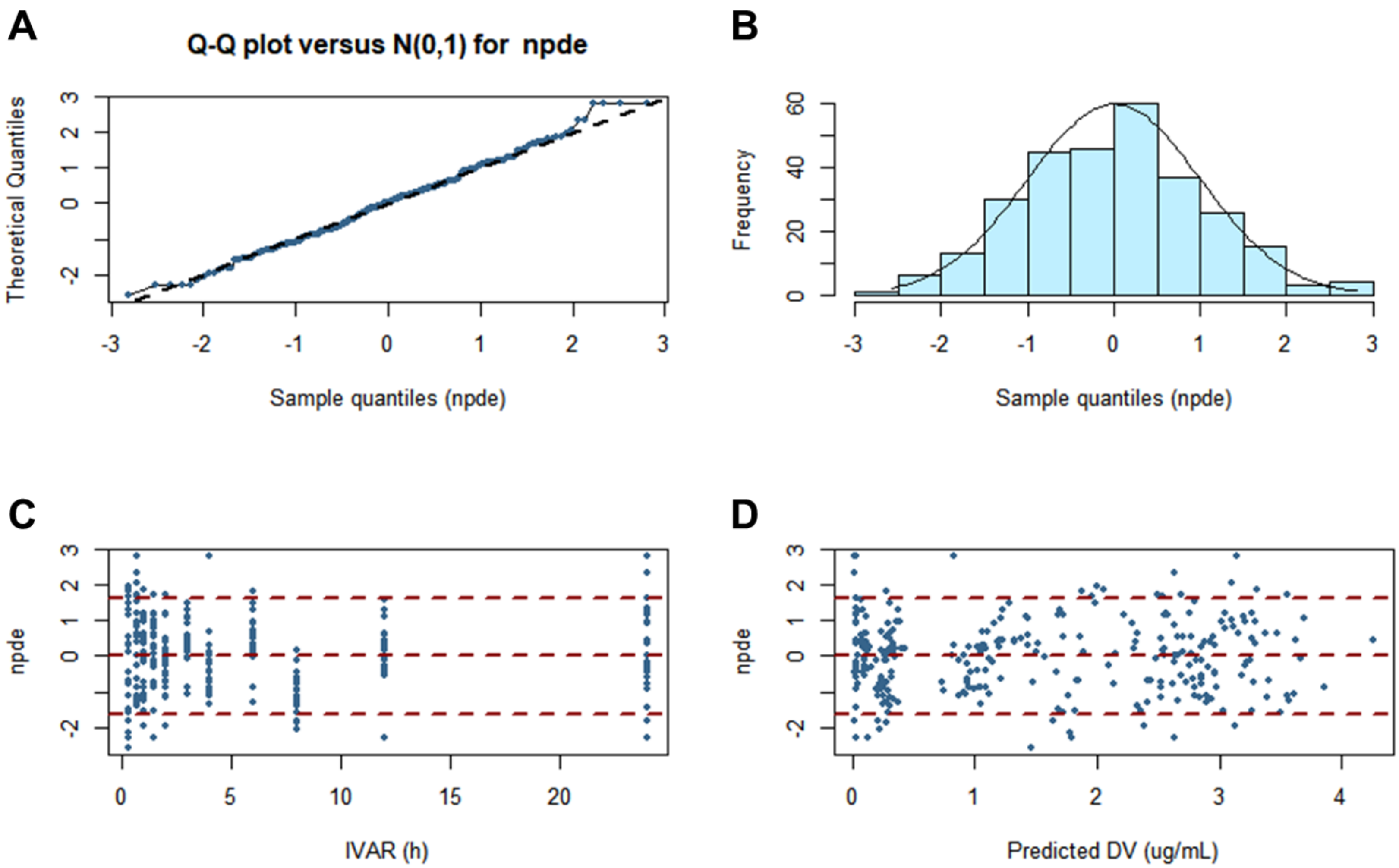
A
Q-Q plot versus N(0,1) for npde
Theoretical Quantiles
Sample quantiles (npde)
B
Frequency
Sample quantiles (npde)
C
npde
IVAR (h)
D
npde
Predicted DV (ug/mL)

Figure S6

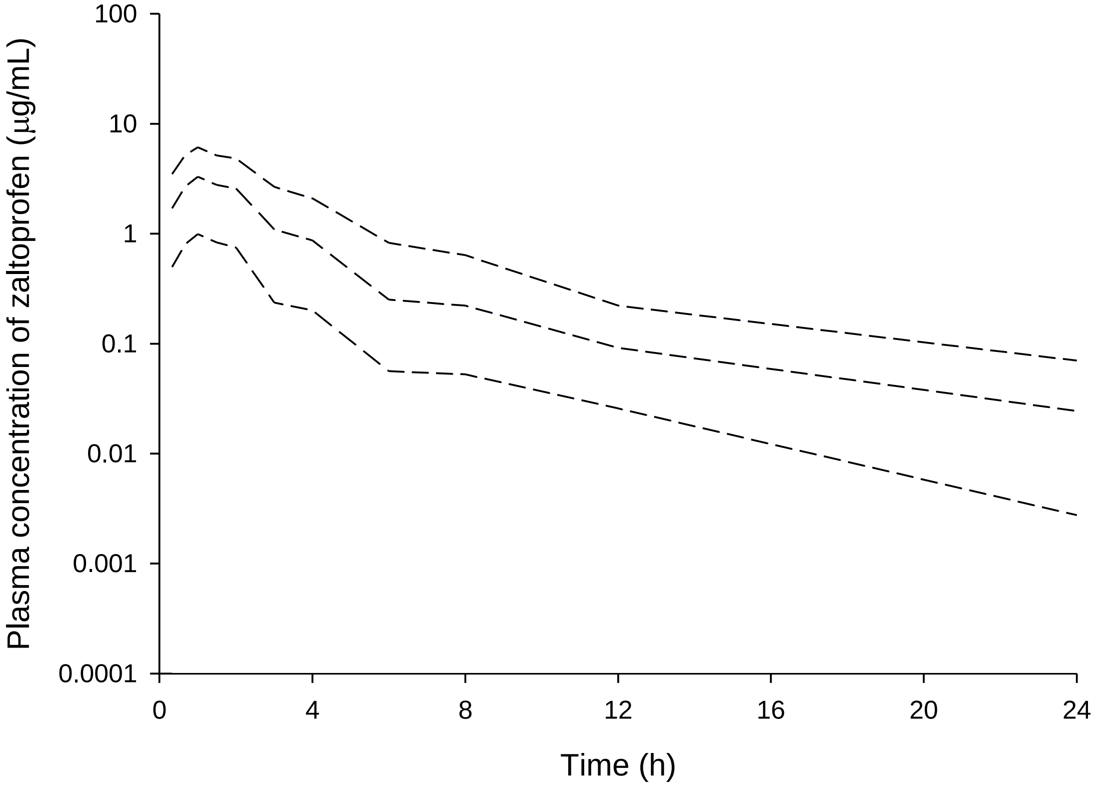

Plasma concentration of zaltoprofen (µg/mL)
100
10
1
0.1
0.01
0.001
0.0001
0
4
8
12
16
20
24
Time (h)

Figure S7

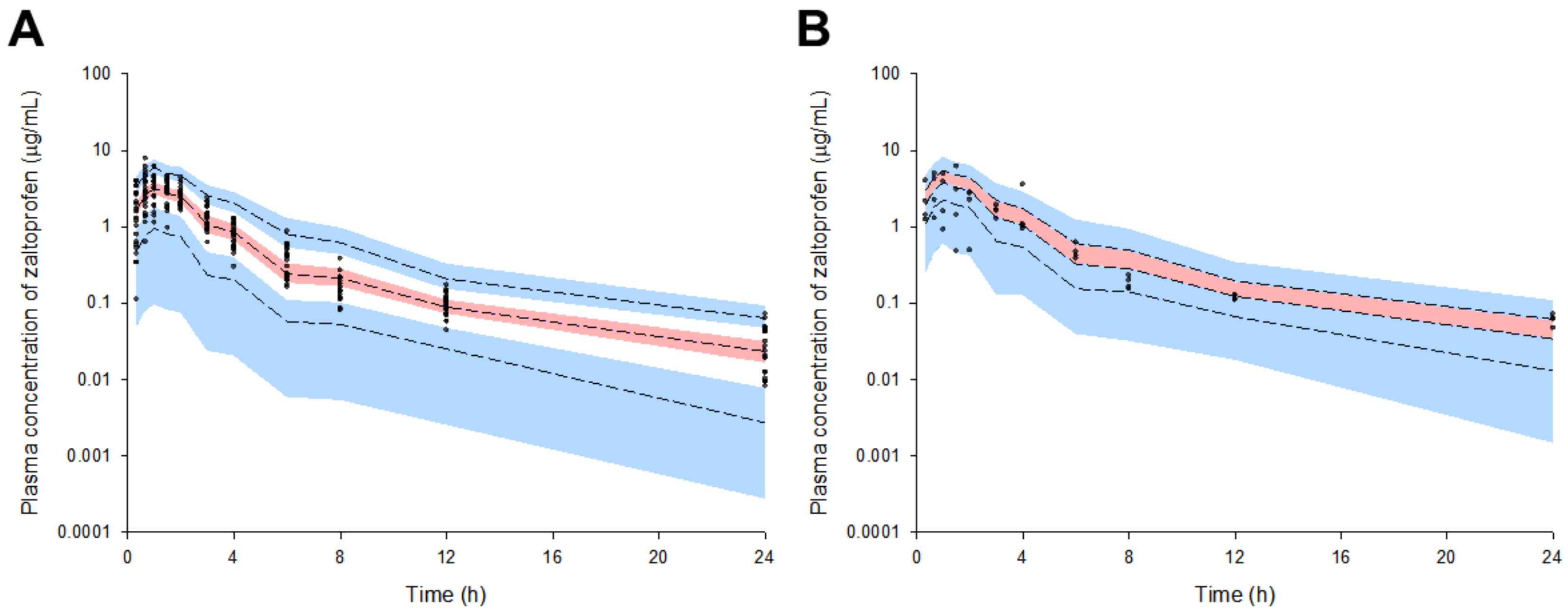
A
B
Plasma concentration of zaltoprofen (µg/mL)
100
10
1
0.1
0.01
0.001
0.0001
0
4
8
12
16
20
24
Time (h)

Figure S8

References

1. Kang, H.-A.; Park, S.; Kim, D.-H.; Kim, H.-H.; Yun, H.; Kim, K.-R.; Yoo, H.-D.; Park, E.-J.; Cho, H.-Y.; Lee, Y.-B. Bioequivalence of SCD Zaltoprofen Tablet to Soleton® Tablet (Zaltoprofen 80 mg). *J Pharm Investig* **2006**, *36*, 209-215.
2. Lee, H.W.; Seo, J.H.; Kim, Y.W.; Jeong, S.Y.; Lee, K.T. Determination of zaltoprofen in human plasma by liquid chromatography with electrospray tandem mass spectrometry: application to a pharmacokinetic study. *Rapid Commun Mass Spectrom* **2006**, *20*, 2675-2680.
3. Li, L.; Ma, P.; Cao, Y.; Tao, L.; Tao, Y. Single-dose and multiple-dose pharmacokinetics of zaltoprofen after oral administration in healthy Chinese volunteers. *J Biomed Res* **2011**, *25*, 56-62.
